# Maternal Interaction Relates to Neural Processing of Self‐Related Multisensory Information in 5‐Month‐Olds

**DOI:** 10.1111/desc.70009

**Published:** 2025-03-18

**Authors:** Nina‐Alisa Kollakowski, Carolina Pletti, Markus Paulus

**Affiliations:** ^1^ Department of Psychology LMU Munich Munich Germany; ^2^ LMU Munich, Graduate School of Systemic Neurosciences (GSN) Planegg Germany; ^3^ Department of Psychology University of Vienna Vienna Austria

**Keywords:** affective touch, attachment, fNIRS, mother‐infant interaction, self‐development, sensitivity

## Abstract

The ontogenetic origin of the self in infancy is a topic of ongoing debate. Although influential developmental and neurocognitive theories propose that caregiver‐infant interactions play an important role in infants’ self‐development, little is known about the specific mechanisms involved. Some theories highlight the importance of caregiver sensitivity and touch, while others propose that caregiver contingency plays a central role. The study aimed to investigate infants’ self‐perception by measuring brain activation in the posterior superior temporal sulcus (pSTS), a region previously associated with self‐related processing. A total of 118 mother‐infant dyads participated in a free‐play interaction, during which maternal sensitivity and touch were measured. Additionally, a face‐to‐face interaction was conducted to measure maternal contingency. Infants' brain activation was measured using functional near‐infrared spectroscopy (fNIRS). They watched a video of their own face while being stroked by a brush on the cheek. The video was either live and the stroking was synchronous to the video (contingent) or the video was delayed by 3 s, which made the stroking asynchronous (non‐contingent). The results showed that infants exhibited more HbO‐activation in the right pSTS in the non‐contingent condition. Importantly, the more sensitive the mothers were and the more they touched infants during free play, the less differential activation the infants showed in response to both conditions. This effect was driven by infants showing less activation to the non‐contingent condition when their mothers exhibited more care, maybe because of a smaller prediction error for non‐contingent self‐related multisensory information. Overall, the study deepens our knowledge of how early social interactions relate to the emergence of the self in infancy.

## 1 Introduction

Based on a renewed interest in the development of the self in infancy (e.g., Ciaunica and Crucianelli 2019; Fotopoulou and Tsakiris 2017; Montirosso and McGlone 2020), the psychological mechanisms subserving the emergence of the self, have become subject to intense debate. One central topic of the theoretical debate concerns whether or not the self develops through social interactions (for recent review see Kollakowski et al. 2023). Although some theories, for example attachment theory (Sroufe 1994) and the theory of mentalizing homeostasis (Fotopoulou and Tsakiris 2017) propose that the social environment is necessary for the infant's self, as it develops out of caregiver‐infant interaction, other theories put less emphasis on social interactions (e.g., sensorimotor theories; Verschoor and Hommel 2017). Previous work on the relation between the caregiver‐child relationship and self‐development focused on the self‐concept and self‐esteem which emerge after the age of 2 (for a review see Harter 2012). However, it has been proposed that an implicit self develops earlier, namely in infancy (Rochat 2009). The implicit self is mostly defined in contrast to self‐concept and self‐esteem, and describes an unreflective, immediate experience of the self that is based on the differentiation of self‐produced and other‐produced perceptual information (for a review see Kollakowski et al. 2023). Little research so far has investigated the extent to which caregiver‐child‐interactions support the development of the implicit self in infancy. This question is particularly interesting given the close relationship between infants and their caregivers. Indeed, developmental theories highlight the existential dependence of infants on their caregivers (e.g., Bowlby 1969), implicating a central role for caregivers in early social and cognitive development. Research on the social basis of the implicit self in these early years is limited though. Therefore, the current study aims at deepening our knowledge on the psychological processes relating to the emergence of the implicit self by investigating relations between the caregiver‐infant interaction and a neurocognitive measure multisensory integration, an ability that is considered as crucial for the developing self (de Klerk et al. 2021).

Attachment theory proposes that the caregiver‐infant relationship plays a crucial role in self‐development (Bowlby 1969). More precisely, it posits that self‐development is a process of organization (Sroufe 1994). Initially, the infant initially is dependent on their caregiver. Then, the infant progressively constructs an internal working model of the caregiver‐infant interaction, becoming ever more capable of organizing themselves independently, which results in increasing psychological and physical separation from the caregiver. This whole process is supposed to lead to the development of the infant's self. The theory posits that infants with a secure attachment exhibit the most stable explicit self, as these infants experienced well‐organized interactions with their caregivers, leading to a well‐organized self (Sroufe 1994). Most empirical investigations of attachment security have primarily focused on the association with self‐esteem, rather than the implicit self, with the majority of the results substantiating the theory (for a review, see Thompson 2008). Yet, it remains an open question to what extent this also holds for the implicit self.

Attachment can only be reliably measured by the end of the first year of life, despite crucial phases of the implicit self‐occurring earlier (Bremner and Spence 2017). Hence, studying early influences of the caregiver‐infant relationship on an infant's implicit self requires different means of investigation, such as predictors of attachment. Caregiver sensitivity, which involves the prompt and accurate identification and response to the infant's signals is a primary predictor of secure attachment (Wolff and van IJzendoorn 1997). Similar to studies on attachment measures, maternal sensitivity measures have been shown to predict self‐esteem and the self‐concept in toddlers (Harel et al. 2002; Harel et al. 1999) and preschool children (Paulus et al. 2018). In a study involving infants, Maister et al. (2020) demonstrated that decreased coordination in mother‐infant interactions increased infants's preference for contingent stimulation when viewing the mother's face. The authors interpreted this preference as a self‐other‐overlap, a state in which seeing another person evokes similar reactions as when viewing oneself. Although the authors did not directly measure maternal sensitivity, highly sensitive mothers are assumed to have more well‐coordinated interactions with their infants, as the mother recognize the infant's signals and adapt to them. Therefore, the results from Maister et al.’s study suggest that infants remain more psychologically and physically attached to the caregiver, when there is a lack of caregiver sensitivity, which is in line with attachment theory. However, Maister et al. investigated self‐other‐overlap, that is, infants’ reactions to pictures of their mothers in contrast to pictures of strangers. It is unclear whether the relation persists when the stimuli presented are self‐related. That is, it remains open and an intriguing question to which extent caregiver sensitivity relates to infants’ developing self. The current study aims at filling his gap.

**Summary**

- Infants exhibit heightened activity in the right posterior superior temporal sulcus (pSTS) when processing non‐contingent self‐related information.
- Maternal sensitivity and touch reduce the difference in neural activity between contingent and non‐contingent conditions.

A different line of developmental theorizing proposes that further caregiver characteristics support an infant's self‐development. Specifically, Bigelow (2001) proposed that contingent interactions between caregivers and infants—defined as the prompt reaction to signals of the infant—train infants to detect contingencies in the environment. This, in turn, helps the infants perceive themselves as detecting contingencies of sensory information, which is a crucial aspect of the implicit self (Botvinick and Cohen 1998; Tsakiris 2008; Weijs et al. 2021). Notably, although caregiver contingency is part of the definition of caregiver sensitivity, it is an independent component of caregiving behavior (Keller et al. 1999). The importance of contingent caregiver‐infant interaction is supported by empirical evidence that demonstrates that an increased number of maternal contingent responses is associated with the infant looking longer at a live video of their own legs compared to a delayed video (Zmyj and Marcinkowski 2017). Also, maternal contingency and maternal imitation are predictors of mirror self‐recognition by the child (Cebioğlu and Broesch 2021; Keller et al. 2005). Consequently, caregiver contingency also seems to help infants to develop an implicit self.

Recent developmental theorizing has offered alternative perspectives on the impact of caregiver‐infant interactions on an infant's implicit self that focus specifically on interpersonal touch (Ciaunica and Fotopoulou 2017; Fotopoulou and Tsakiris 2017; Montirosso and McGlone 2020). Precisely, it has been proposed that tactile interactions between caregivers and infants aid caregivers in adapting to their infant's needs, consequently increasing the caregiver's sensitivity (Montirosso and McGlone 2020). Furthermore, tactile interactions are assumed to contribute to the development of an implicit self in infants by providing sensory information about their bodies (Ciaunica and Fotopoulou 2017). Although this line of theorizing considers all physical contact to be contributing to self‐development, they highlight the role of affective touch, which has a stroking or caress‐like quality, as it also communicates emotional information. Studies with adults have shown that affective touch can enhance self‐perception (Crucianelli et al. 2013; Lloyd et al. 2013; van Stralen et al. 2014). Furthermore, affective touch is a significant predictor of attachment (Woodhouse et al. 2020), indicating that it might be especially important for the infant's developing self. Notwithstanding increased interest in the role of touch, there is little empirical evidence on whether caregiver touch affects the development of an infant's self (see Della Longa et al. 2019 for preliminary evidence).

In summary, various theoretical approaches predict that characteristics of caregiver‐infant interaction, such as sensitivity, contingency, and touch, play a significant role in infants’ self‐development. The infant's self has usually been investigated by measuring their looking preference to contingent or non‐contingent self‐related information (e.g., Bahrick and Watson 1985; Filippetti et al. 2013; Rochat and Morgan 1995). However, recent research has also investigated neural activation patterns during tasks involving contingent and non‐contingent information (Bulgarelli et al. 2019; Filippetti et al. 2015). The basis for this research line were studies with adults that demonstrated the involvement of various brain regions, predominantly located in the right hemisphere and parietal cortices (for reviews, see Blanke 2012; Gillihan and Farah 2005; Tsakiris 2010). The right temporo‐parietal junction (TPJ) is a brain region that frequently exhibits activity in self‐related processing (Quesque and Brass 2019). Also, research with infants highlights the involvement of these brain regions in the infant's self: Bulgarelli et al. (2019) found that infants who recognized themselves in mirrors at 18 months exhibited more connectivity between the medial prefrontal cortex and the right TPJ than those who did not yet show mirror self‐recognition. Filippetti et al. (2015) demonstrated that 5‐month‐old infants had increased brain activity in the bilateral posterior superior temporal sulcus (pSTS) and left inferior frontal gyrus while viewing live video footage of their own face brushed compared to watching a delayed recording.

Interestingly, also the caregiver characteristics presented herein are linked to the development and activation of these particular brain areas. It has been revealed that attachment style relates to the activation of a mentalization brain module, including pSTS (Long et al. 2020). STS and TPJ also exhibited greater activation during caregiver‐infant interactions that were contingent, in contrast to pre‐recorded or non‐contingent interactions (Hakuno et al. 2020; Hakuno et al. 2018; Lloyd‐Fox et al. 2015; Piazza et al. 2020). Likewise, social touch activates a broad network of brain regions, such as the pSTS (Björnsdotter et al. 2014; Brauer et al. 2016). However, there is conflicting evidence regarding the neural distinction between affective and non‐affective touch in infancy (Jönsson et al. 2018; Pirazzoli et al. 2019). In accordance with the studies reviewed above, some researchers claim that the pSTS is a region responsible for social cognition (Deen et al. 2015). Especially, the pSTS seems to be involved in theory of mind, processing of biological motion and faces (see Hein and Knight 2008, for a review). However, others claim that the involvement of the pSTS in these functions can also be explained more parsimoniously by its involvement in multisensory integration (Beauchamp 2015). In any case, the pSTS seems to be a fitting target region for our study's aim.

## 2 Current Study

This study aims to investigate the origin of the self during infancy. Specifically, we examine whether caregiver‐infant interaction plays a role in forming the infant's implicit self. Various theories, like attachment theory, predict that facets of caregiver‐infant interactions aid infants in developing their implicit self. Consequently, higher levels in the investigated caregiver characteristics should positively predict the measures of the infant's self.

The present study aims to provide an empirical touchstone for influential theories on the relation between caregiver‐infant interaction and infants’ developing self. Based on three theoretical approaches, three different measures are considered, namely caregiver sensitivity, contingency, and touch. Measuring all of the different interaction characteristics in one study gave us the opportunity to assess whether each explains unique variance in the infant's self‐development. Caregiver sensitivity was measured using the Emotional Availability Scales (Biringen 2008). Within the Emotional Availability Scales, sensitivity is defined as a dyadic construct in which caregiver and infant exert bidirectional influences on each other. Therefore, the measure gives a holistic picture of the caregiver‐infant interaction, from which the infant's self develops according to attachment theory. Caregiver contingency was assessed in a face‐to‐face interaction, as in previous studies (e.g., Keller et al. 1999; Zmyj and Marcinkowski 2017). Two types of caregiver touch were assessed. Although theories emphasizing the role of interpersonal touch on the developing self hypothesize all types of touch to be beneficial for self‐formation, a special role is ascribed to affective touch, transporting also emotional information. However, so far, little research has confirmed a special role of affective touch over touch in general (Della Longa et al. 2019). The current study, therefore, considered interpersonal touch in general, independent of the type of touch, and affective touch to investigate whether affective touch indeed plays a special role beyond general touch.

Infants’ self‐development was measured with functional near‐infrared spectroscopy (fNIRS). Previous research with adults demonstrated that temporal and parietal areas are consistently activated by self‐related information. A study by Filippetti et al. (2015) that investigated whether infants show a similar activation pattern demonstrated activation in a region spanning the pSTS. According to Filippetti et al. (2015), the heightened pSTS activation while watching a live video of the infant's face being stroked by a brush indicated more integration of the contingent multisensory information. Integration of multisensory information is an integral part of self‐development (de Klerk et al. 2021). Thus, the more pSTS activity infants show while watching the live video, compared to a delayed video, the more they manage to integrate the multisensory information, indicating a more pronounced self‐development. Based on these findings, in the current study, we hypothesized higher scores on the caregiver characteristics to be related to more activation to the contingent condition compared to the non‐contingent condition in the pSTS.

## 3 Methods

### 3.1 Participants

Altogether 118 mother‐infant (54 infants female) dyads participated in the study. The mean age of the infants was 5 months and 19 days (SD = 13.69 days) at the behavioral testing session. fNIRS was measured in a separate session, on average 8 days (SD = 10) after the behavioral session, in which 98 infants participated. Mothers were on average 34 years old (SD = 3.46). One mother did not disclose her age. All infants were typically developed and born after 37 weeks of gestation. Families were recruited from a large city in Germany via public birth records, therefore the sample was mainly from middle‐class white background. Mothers gave informed consent for their and their infants participation in the study and received compensation for travel expenses and a small gift for the infant. The study was approved by the department's ethical committee. Testings took place between July 2020 and April 2021.

### 3.2 Behavioral Testing Session

#### 3.2.1 Procedure

Upon arrival, mothers were informed about the study procedure, and infants were given some time to get accustomed to the environment. Then, the infants were placed into the infant seat, facing the mother. The experimenter left the room for 8 min while the mother and infant were interacting face‐to‐face. Subsequently, mother and infant moved to the floor for the free play‐situation, for which the experimenter again left the room for 10 min in total. After 5 min, the experimenter re‐entered the room to make toys available to the dyad. At the end of the session, the experimenter measured the infant's head circumference for the fNIRS session that took place at another session a few days later. Given the pandemic circumstances, mothers and experimenters wore face masks when in the same room at the same time. Mother‐infant interactions were conducted without masks, or if mothers refused to take off their masks, these data were excluded.

##### 3.2.1.1 Maternal Contingency

The setup followed Zmyj and Marcinkowski (2017). Infants lied in an infant seat that was mounted on a table. The mother sat exactly in front of the table on a chair facing the infant. One camera was located behind the infant to record the mother's face, while the other camera was placed next to the mother to film the infant. Mothers were instructed to interact normally with the infant for 8 min but without using any objects or toys and while leaving the infant in the infant seat. Before leaving the room, the experimenter touched the infant's seat as a visual cue to synchronize both videos for analysis.

##### 3.2.1.2 Maternal Sensitivity and Maternal Touch

Mothers and infants sat on a blanket on the floor. Cameras stood at opposite sides of the blanket to film the interaction. Mothers were instructed to interact normally with the infant for 10 min—the first 5 min, they were not allowed to use any objects or toys, then the experimenter made age‐appropriate toys available.

#### 3.2.2 Coding

All tasks were coded using Datavyu version 1.3.7 (Datavyu Team 2014). For each task, a trained assistant coded the data of the whole sample, while a second trained and independent assistant coded 20% of the data. Inter‐rater reliability measures were calculated between these two coders.

##### 3.2.2.1 Maternal Contingency

The coding followed Zmyj and Marcinkowski (2017). The first minute of the interaction was considered a warm‐up phase and therefore not analyzed. From then, 4 min of the interaction were coded as this decreased the drop‐out rate compared to coding the entire interaction. At first, the coder identified episodes of mutual gaze, which were then inspected further. In separate passes the coder recorded the verbal and non‐verbal utterances of the mother and infant. Then, the facial expressions of mother and infant were coded separately. Three facial expressions were considered: smiling, lifting or scrunching the eyebrows, and tongue protrusion. For the mother, smiling was coded when the corners of the mouth were at least above the middle of the mouth. For the infant, smiling was coded when the corners of the mouth were at least parallel to the middle of the mouth. Tongue protrusion was coded when the tip of the tongue passed the lips. Reliability was computed using GSEQ (Generalized Sequential Querier; Bakeman et al. 2009), which computes two forms of Cohen's kappa: the time‐unit kappa comparing the timing of each coder's sequences, and the event‐alignment kappa comparing the event sequences of both coders. For the time‐unit kappa we tolerated 1 s of deviation; for the event‐alignment kappa 2 s of deviation were tolerated; however, the events had to overlap 80% of the time. The time‐unit kappa was 0.92–0.92 (agreement: 97%–97%), the event‐alignment kappa was 0.62 (76% agreement).

Each maternal behavior that started within 1000 ms after the onset of the infant's facial or vocal expression was considered a contingent behavior. For the infant's vocal expressions, maternal behavior was still considered contingent when it started within 1000 ms after the offset of the infant's expression, to also include mothers that did not want to interrupt their infant. If the maternal behavior started at the same time as the infant's behavior, it was not considered contingent. If two maternal behaviors fell within the latency window, only the first one was considered as contingent. If the infant produced several behaviors simultaneously, the maternal behavior was considered contingent to each of them separately.

Next, we computed a responsiveness index for maternal contingency that takes the total amount of maternal behavior into account (Keller et al. 1999; Equation 1). This way, the responsiveness index indicates whether the mother was contingent to the infant's behavior above chance level:

(1) $$\frac{\mathrm{maternal}\;\mathrm{contingent}\;\mathrm{behaviors}}{\mathrm{infant}\;\mathrm{behaviors}}-\left(1-\;e^{\frac{\mathrm{maternal}\;\mathrm{behaviors}}{\mathrm{duration}\;\mathrm{of}\;\mathrm{mutual}\;\mathrm{gaze}}*1000}\right)$$

The two‐way mixed model ICC was 0.65 for the responsiveness index between coders. Data from 97 dyads were analyzed as 7 were excluded because of technical errors, 4 were excluded because the mother refused to take off her mask, and 10 because of fussiness of the infant.

##### 3.2.2.2 Maternal Touch

From the free‐play interaction, the first 5 min without toys were analyzed for this measure. The video was divided into 2 s‐long segments. For each segment, the coder decided for one of seven categories of touch, which were derived from Crucianelli et al. (2019) and Provenzi et al. (2020): affective, harsh, playful, attention‐getting, instrumental, static, and incidental touch (see Table 1 for definitions). If more than one touch category was present within the 2‐s‐segment, the category that appeared earlier in the enumeration above was coded, as we considered the more intentional and more affective touches more important. Only touches performed with the hand were coded. When no touch occurred within a segment, no touch category was coded. Coders initially discussed three videos for training before coding each of their datasets individually. Cohen's Kappa for the coding was 0.78.

**TABLE 1:** Codes of maternal touch used in this study.

Category	Definition
Affective	Intentional, low‐energy, and slow touch that gives a sense of closeness between mother and child
Harsh	Intentional touch that is not contingent with the infant's emotional state by being too fast or intense for the infant
Playful	Intentional, fast‐paced touch with the goal to make the infant smile or laugh
Attention‐getting	Intentional touch with the aim to direct the infant's attention to the mother, for example, by tapping on the infant
Instrumental	Intentional touch without a communicative value, intending to change or maintain the infant's position, clean the infant, or adjust the infant's clothes
Static	Mother keeping her hand in contact with the child without moving it, intending to maintain physical contact
Incidental	Mother touching the infant when actually aiming at something else

Subsequently, a total duration was computed for each category. We assumed that each touch occurred for the whole duration of the 2s‐segment. For the general touch variable, a sum was built over all touch categories, representing the total amount of time spent with the mother touching the infant. To account for slightly different interaction lengths, the absolute time of all touch categories and affective touch was divided by the total duration of the video. The ICC for these measures was 0.98 between coders. Data from 113 dyads was analyzed. Data from 4 dyads had to be excluded because of experimental errors, data from 1 dyad because of a technical error.

##### 3.2.2.3 Maternal Sensitivity

Maternal sensitivity was coded according to the Emotional Availability Scales 4.0 (Biringen 2008) using the entire 10 min of free‐play interaction. According to the coding manual, sensitivity is scored on a 7‐point scale, representing a holistic score for the whole session. A 7 indicates a highly sensitive caregiver, a 4 represents inconsistent sensitivity, a 3 indicates somewhat insensitive caregivers, and a 1 represents highly insensitive caregivers. To receive a high score on this scale, caregivers need to display a warm affect, clear perceptions of the infant's signals and appropriate responses to these signals, have a good awareness of timing, flexibility in their behavior, display respect in their interaction with the child, as well as a smooth handling of conflict situations. Both coders completed the necessary training for coding and achieved acceptable reliability before coding the present dataset. Reliability on the present dataset was ICC = 0.86. 117 datasets were analyzed as 1 dyad had to be excluded due to technical issues.

### 3.3 fNIRS Session

#### 3.3.1 Procedure

Upon arrival, caregivers and infants were brought to a sound‐attenuated, dimly lit, and electrically shielded testing booth. The caregiver was informed about the procedure and the functioning of the fNIRS system, while the infant had some time to get accustomed to the environment. The infant sat on the caregiver's lap approximately 70 cm away from a computer screen positioned at eye‐level of the infant. A webcam (Logitech C920 HD Pro) was positioned directly below the computer screen to film the infant's face. Caregivers were instructed to refrain from interfering with the task by avoiding movements and talking. Given the pandemic circumstances, mothers and experimenters wore face masks during the whole session.

**FIGURE 1:**
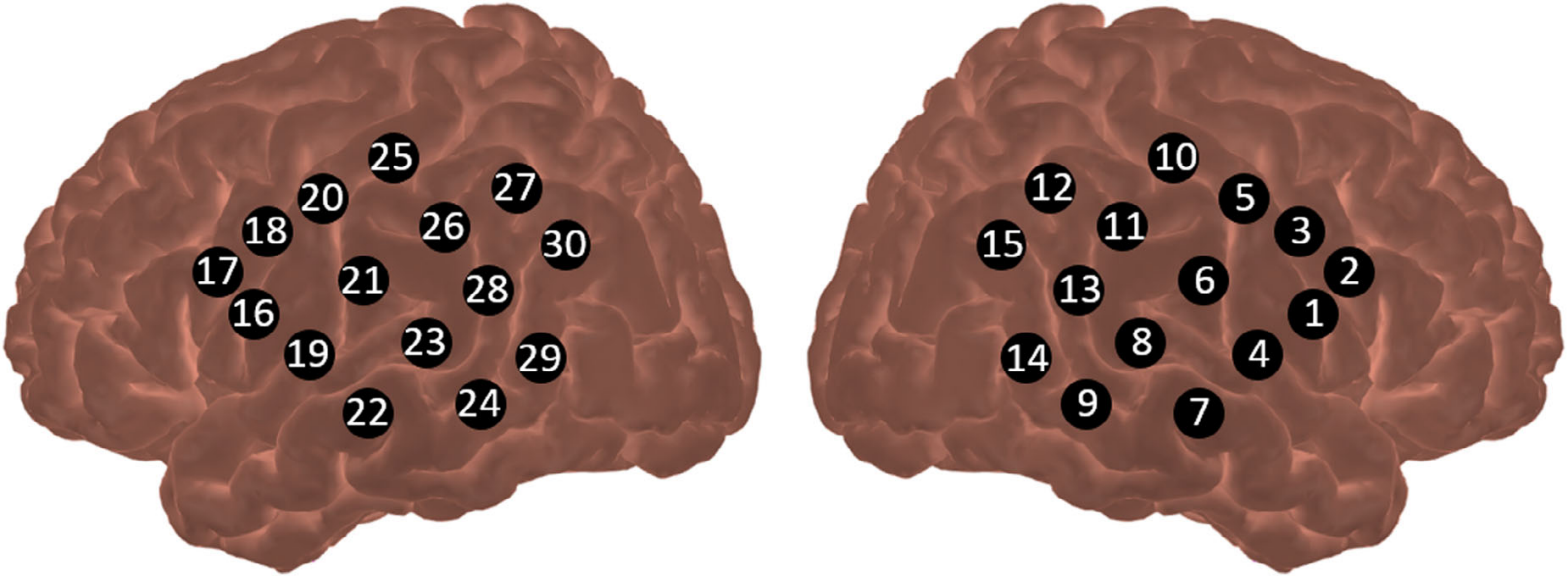
fNIRS channel placement on the left and right hemispheres. *Note*: Each number denotes a channel, lying in the middle between source and detector.

Two experimenters fitted the fNIRS cap on the infant while the infant was watching videos on the screen. The fNIRS was a NIRScout (NIRx Medical Technologies GmbH, Berlin, Germany) and had 8 sources and 16 detectors, measuring 30 channels in total with two continuous wavelengths at 760 and 850 nm. The channels were located temporally on the left and right of the infant's head. The optodes were placed according to the 10‐5 system (Oostenveld and Praamstra 2001) to ensure a source‐detector separation between 20 and 25 mm. Sources were located at C5, C6, TP7, TP8, CP3, CP4, P5, and P6. Detectors covered the locations FC5, FC6, FCC5h, FCC4h, FTT7h, FTT8h, T7, T8, C3, C4, CP5, CP6, P7, P8, P3, and P4. As the measured brain area lies approximately in the middle of the source and detector, this results in the channel locations in Figure 1.

After the calibration of the fNIRS, the testing booth door was closed. One experimenter remained within the booth, sitting on the left side of the caregiver, controlling the task, and applying the brush strokes to the infant. This experimenter was the same for all infants. The other experimenter controlled the fNIRS recording outside the booth. The fNIRS was recorded with NIRStar 15.2 (NIRx Medical Technologies GmbH, Berlin, Germany).

The task followed the procedure of Filippetti et al. (2015) and consisted of two conditions with up to 10 trials each: in the contingent condition, infants saw a live video of their own face on the screen. In the non‐contingent condition, the video was delayed by 3 s. Each trial lasted 15 s and was preceded by a 12 s baseline, in which six different pictures of vehicles were presented with a pseudo‐randomized duration of 1, 2, or 3 s. The videos and pictures were presented by OpenSesame version 3.2.8 (Mathôt et al. 2012), with the OpenCV library for video recording and presentation. During the video presentation in the trials, the experimenter brushed the infant's left cheek a maximum of three times per trial (contingent: *M* = 2.77, SD = 0.38; non‐contingent: *M* = 2.76, SD = 0.45; *t*(82) = 0.27, *p* = 0.79). Each brushstroke lasted 695 ms (SD = 136) on average in the contingent condition and 667 ms (SD = 126) in the non‐contingent condition (*t*(82) = 3.15, *p* = 0.002). In the non‐contingent condition, the experimenter took care that the actual brushstroke did not happen at the same time as the visible brushstroke. The order of conditions followed an ABBABAAB rhythm, starting with the contingent condition.

During the whole task, piano music played to calm down the infants. Whenever the infant was fussy, the experimenter could initiate a break. When the infant was too fussy to continue, the task was aborted. To draw back the infant's attention to the screen, the experimenter could play a sound at the beginning of the baseline. In these cases, the same sound was also played at the beginning of the respective trial.

#### 3.3.2 Pre‐Processing

Data of 14 infants had to be excluded before pre‐processing because of technical issues (8) or fussiness (6). The processing was conducted in Homer2 (Huppert et al. 2009). First, trials were rejected based on the looking time of the infant: only trials in which the infant looked to the screen more than 50% of the trial time and looked at the screen for at least 50% while the brushstrokes were applied, were included. A minimum of three valid trials per condition was necessary for the infant to be included in further analysis; 28 infants did not meet these criteria. The remaining infants, on average, completed 6.19 trials (SD = 1.97) in the contingent condition and 5.87 trials (SD = 2.04) in the non‐contingent condition (*t*(61) = 1.80, *p* = 0.08). Data from the remaining trials were first checked for channels whose activity was outside of a range from 0.09 to 1 V or whose signal‐to‐noise ratio was smaller than 0. Data were then converted to optical density. Following recommendations from Di Lorenzo et al. (2019), we then removed motion artifacts by applying a sequence of first detecting motion artifacts by channel with a change in a channels’ signal of more than 14 standard deviations or more than 0.4 optical density units in amplitude within 1s marked as motion artifacts. These motion artifacts were then corrected with wavelet filtering with an interquartile range of 0.5. Subsequently, we wavelet‐transformed the HbO function for each channel and visually checked the channels for a visible heart rate and motion artifacts (see Nguyen et al. 2021). Channels were excluded if they showed no heart rate or too many motion artifacts. Finally, data were again checked for remaining motion artifacts. Contaminated trials were then rejected. Afterward, data were bandpass filtered between 0.01 and 1 Hz and converted to concentration using the modified Beer–Lambert law with a differential pathlength factor of 5.1. Two regions of interest (ROI) were formed, both spanning the superior temporal sulcus on both hemispheres. The ROI on the right hemisphere included channels 8, 9, 13, and 14. The ROI on the left hemisphere included channels 23, 24, 28, and 29. The signal from all channels within an ROI was averaged for subjects that had data for at least 2 channels within an ROI.

Overall, 57 subjects contributed data to the left ROI with an average of 3.26 channels (SD = 0.81) per subject. Fifty‐four subjects contributed data to the right ROI with an average of 3.11 channels (SD = 0.86) per subject.

### 3.4 Data Analysis

All analyses were conducted in RStudio. To check for relations between the maternal variables, we used Pearson product‐moment correlation.

For analysis of the fNIRS data, we followed de Klerk et al. (2018). Six time bins were created (3 s each) of the post‐stimulus time window, in which data were averaged for each ROI and condition separately. A repeated‐measures ANOVA was conducted, using time bins and conditions as within‐subject factors. This way, we analyzed whether there was a significant change of activation over time and whether this activation differed significantly between conditions.

For each ROI, we then computed difference scores, averaging the signal over the whole post‐stimulus time range and subtracting the signal in the non‐contingent condition from the signal in the contingent condition. These difference scores were then used as dependent variables in a linear regression model. Independent variables were the relative amount of time mothers touched their infants, the relative amount of time spent with affective touch, the maternal sensitivity score, the contingency responsiveness index, and the age of the child at the time of the fNIRS session.

## 4 Results

Descriptive statistics of all maternal variables, as well as correlations between maternal variables are reported in Table 2. Only maternal touch in general and maternal affective touch were significantly correlated.

**TABLE 2:** Descriptive statistics and correlations of maternal variables.

Variables	*n*	*M*	SD	1	2	3
1. Sensitivity	117	5.08	1.11	—		
2. All touch	113	62.19%	27.12	−0.02	—	
3. Affective touch	113	10.38%	9.24	0.15	0.41^***^	—
4. Contingency	97	−0.12	0.26	0.10	0.07	0.13

^*^ *p* < 0.05.

^**^ *p* < 0.01.

*** *p* < 0.001.

### 4.1 fNIRS Analysis

**FIGURE 2:**
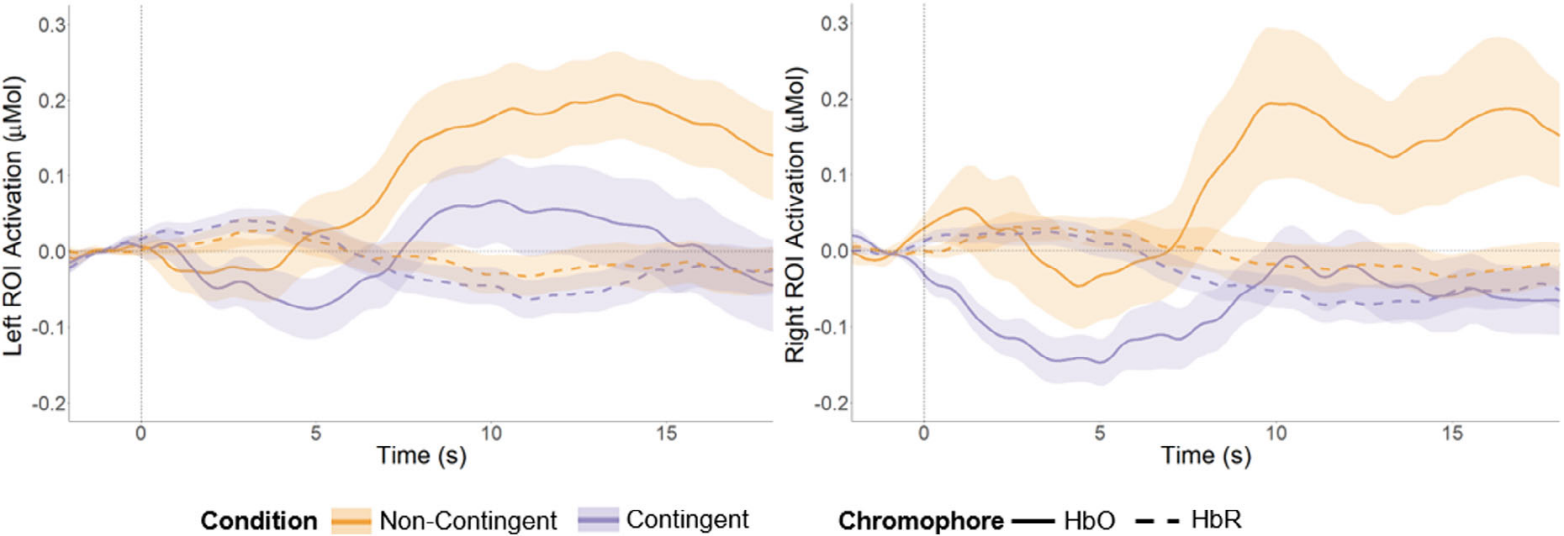
Grand averages of fNIRS signal over both regions of interest. *Note*: Grand averages over the region of interest (ROI) on the left hemisphere (left) and on the right hemisphere (right). Averages of the contingent condition are presented in orange. Averages of the noncontingent condition are presented in purple. Solid lines represent the oxygenated hemoglobin concentration (HbO), while dashed lines indicate the concentration of deoxygenated hemoglobin (HbR). Although the left ROI shows no significant differences between the conditions, HbO differences in the right ROI are significant over the whole time range.

The left ROI (see Figure 2) showed a significant difference in HbO‐activation over time, indicated by a main effect of time bin (*F*(5, 275) = 9.35, *p* < 0.001). Also, the interaction effect between condition and time bin was significant (*F*(5, 275) = 3.02, *p* = 0.01), but the main effect of condition was not significant (*F*(1, 55) = 2.92, *p* = 0.09). However, post‐hoc *t*‐tests on each time bin revealed no significant differences between both conditions in HbO concentration (all *p* > 0.05; see Table S2). Analyses of HbR in the left ROI revealed a significant difference in activation over time, that is a main effect of time bin (*F*(5, 275) = 7.62, *p* < 0.001) but no other significant effects (all *p* > 0.43). The difference score for the left ROI had an average of −0.09 µMol (SD = 0.41).

The right ROI showed a significant difference in HbO‐activation over time (*F*(5, 260) = 10.10, *p* < 0.001) and a significant main effect of condition (*F*(1, 52) = 6.08, *p* = 0.02). Post‐hoc *t*‐tests (see Table S1) on each time bin revealed significant differences in the whole time range with more activation in the non‐contingent condition than in the contingent condition (see Figure 2). Analyses of the HbR in the right ROI revealed significant activation differences over time (*F*(5, 260) = 10.59, *p* < 0.001) but no other significant effects (all *p* > 0.25). The average difference score for the right ROI was −0.15 µMol (SD = 0.44). Differences between the conditions cannot be explained by differences in looking time (see Table S6).

### 4.2 Regression Models

The linear regression model with the differential activation in left ROI as the outcome variable was not significant (*F*(5, 380) = 0.51, *p* = 0.77, adjusted *R^2^* = −0.06). No predictor had a significant effect on the differential activation in the left ROI (see Table S3).

The linear regression model with the differential activation in the right ROI activation as the outcome variable was significant (*F*(5, 35) = 3.88, *p* = 0.007, adjusted *R^2^* = 0.26). Maternal sensitivity as well as the total amount of maternal touch, were significant predictors (see Table 3). As the ROI difference score indicated how much more activation there was to the contingent condition (compared with the noncontingent condition), and since the difference score was negative, the positive estimates of both predictors mean that more maternal touch and more maternal sensitivity lead to less differential activation in the right ROI.

**TABLE 3:** Linear regression results on the differential activation in the right ROI.

Variable	Estimate	SE	95% CI	*t*‐value	*p* value
**Intercept**	**−2.1242**	**0.7053**	**[−3.5561, −0.6922]**	**−3.01**	**0.005**
Affective touch	−0.0049	0.0079	[−0.0208, 0.0111]	−0.62	0.54
**All touch**	**0.0062**	**0.0023**	**[0.0014, 0.0109]**	**2.65**	**0.01**
Contingency	−0.1297	0.2543	[−0.6461, 0.3866]	−0.51	0.61
**Sensitivity**	**0.1382**	**0.0556**	**[0.0253, 0.2512]**	**2.49**	**0.02**
Age	0.0051	0.0038	[−0.0026, 0.0129]	1.35	0.19

*Note*: Significant (*p* < 0.05) variables are presented in bold font.

Post‐hoc tests revealed that more maternal sensitivity and maternal touch led to less activity in the non‐contingent condition (see Table S4), while there was no significant influence of any predictor on the activity in the contingent condition (see Table S5).

## 5 Discussion

Developmental theorizing has a long‐standing interest in understanding how caregiver interactional characteristics relate to an infant's developing self. Influential developmental theories propose that sensitive and contingent caregiving, as well as (affective) touch during interaction, promote the development of the self in infancy. The current study investigated theoretical claims that these characteristics would positively predict the infant's ability for multisensory integration, an important precursor to the self (de Klerk et al. 2021). Multisensory integration was measured with the infant's brain activity in the pSTS while watching contingent videos of themselves, compared to non‐contingent videos. In contrast to our assumption, infants showed more activity in response to the non‐contingent condition. Importantly, maternal sensitivity and maternal touch negatively predicted the infants’ brain activity to the non‐contingent condition. This suggests a relation between maternal interaction characteristics and neurocognitive processes that support infants’ developing self.

Interestingly, contrary to a previous study (Filippetti et al. 2015), infants in our study showed more right pSTS activity in response to the non‐contingent condition than to the contingent condition. At the same time, studies with adults demonstrated similar patterns as reported in our study (e.g., Ionta et al. 2011; Kontaris et al. 2009). For instance, a study by Leube et al. (2003) manipulated the delay between participants' hand movement and the visual feedback of the movement. This study found a positive correlation between the temporal delay and the activity in the right pSTS. Typically, increased activation to non‐contingent information is interpreted as a prediction error in these studies (e.g., Kontaris et al. 2009). Participants predict that visual feedback of their bodily sensations, such as touch or movement, will be temporally contingent with these sensations. If this is not the case, a prediction error arises, which is represented by higher neural activation. The existence of prediction errors in infancy has been repeatedly demonstrated (Berger and Posner 2023). Therefore, our discovery that infants exhibit heightened neural activity in response to the non‐contingent condition could be interpreted as a prediction error in relation to visual information that does not correspond to their bodily sensations. The emergence of a prediction error for non‐contingent visual information suggests that infants have an expectation that visual information will match their bodily sensations. Previously, the establishment of these expectations was considered to be the beginning of the infant's implicit self (Rochat 1998). Thus, higher neural activation to the non‐contingent condition compared to the contingent condition would indicate a more pronounced implicit self. This result supports developmental theories that highlight the impact of predictive processes on the development of the self (see Kollakowski et al. 2023 for a recent review).

It is noteworthy that increases in maternal sensitivity and touch are associated with less neural differentiation between conditions, indicating that the response to both conditions is more similar. This effect is primarily due to increases in maternal sensitivity and touch being associated with reduced activation in the non‐contingent condition, resulting in less prediction error. Both measures, however, are not correlated, indicating that they are independent of each other, although both contribute to infant attachment (Anisfeld et al. 1990; Biringen et al. 2014). Indeed, both might fulfill different functions for infants’ self‐development. Maternal touch, as measured in this study, represents the number of touches delivered to the infant, independent of the function and appropriateness of the specific touch. Thus, this measure assesses the amount of tactile (and maybe visual) information delivered to the child. According to the theory of mentalizing homeostasis (Ciaunica and Fotopoulou 2017; Fotopoulou and Tsakiris 2017), this would represent a learning opportunity for multisensory integration, an important factor in self‐development. Although the present findings support the theory of mentalizing homeostasis in their claim that interpersonal touch plays a role in self‐development, more touch leads to a less pronounced self‐development. Consequently, it might be the case that the not perfectly contingent information provided by the mother rather trains the infant to have a broader time range for considering information as contingent. Therefore, they show less prediction errors within the non‐contingent condition. Indeed, previous studies have shown that a delay of 3 s might be right at the threshold for perceiving information as contingent or non‐contingent, at least for infants in their first 6 months of life (Gergely and Watson 1999; Rochat and Striano 2000; Zmyj et al. 2009). Although our results, on a group level, show that neural activation is different between the non‐contingent and contingent condition, indicating that infants in this study differentiated between both, it is possible that especially infants from mothers who touched their infants more did not perceive this difference in contingency.

Despite recent theories emphasizing the significance of affective touch in the caregiver‐infant interaction (Ciaunica and Fotopoulou 2017; Montirosso and McGlone 2020), our findings indicate that there is no specific contribution of affective touch beyond touch in general to the infant's self. Currently, only one study has demonstrated a specific role of affective touch in behavioral measures of contingency detection in infancy (Della Longa et al. 2019). In contrast, our study examines the neural activation of infants' brains under different contingency conditions. Previous studies have shown mixed evidence regarding the differences in brain activation between affective and non‐affective touch in infancy (Jönsson et al. 2018; Pirazzoli et al. 2019). Therefore, it is possible that neural and behavioral measures are differentially affected by affective touch or touch in general.

Maternal sensitivity goes beyond maternal touch in that it also provides the infant with sensory information mostly contingent to their own signals, but in this measure also the appropriateness of the reaction to the infant's signal is considered. According to attachment theory, the implicit self results from a well‐organized caregiver‐infant interaction, hypothesizing that appropriate reactions to the infant's signals result in a more organized interaction. Consequently, increases in maternal sensitivity are hypothesized to promote an infant's implicit self, which our results contradict. One possible explanation for this finding is that a more pronounced self is not beneficial for the infant, at least at this age. Verschoor and Hommel (2017) for example hypothesize that a less pronounced self and, therefore, less self‐other differentiation is beneficial for observational learning. If the actions of another person are considered as their own actions due to a lack of differentiation between self and other, infants could incorporate these actions into their action repertoire without the need of establishing a correspondence between themselves and the other. Thus, infants should be less sensitive to imperfect contingencies of multisensory information, as sensory information produced by other people will be less contingent than self‐produced sensory information, and therefore, these infants should produce less prediction error when seeing non‐contingent information. A similar idea has been put forward by Lewis et al. (1985) and Mahler et al. (1985). Lewis et al. (1985) demonstrated that children with insecure attachment styles showed earlier mirror self‐recognition than children with secure attachment styles, Children with less aligned caregivers, resulting in insecure attachment styles, in contrast might need to develop a self earlier to be able to act on their own, compensating for a lack of care.

Although maternal contingent responsiveness has been hypothesized to influence the infant's self (Bigelow 2001), we did not find evidence for this relation in our study. There could be several reasons for this. Firstly, our measure of maternal contingency was limited to facial expressions and vocal utterances of the mother and child and did not incorporate movements of other body parts. This may have provided a too‐limited view of contingency. Furthermore, other theories suggest that caregiver contingency has an impact on affective self‐regulation (Gergely et al. 2010) or agency (Sroufe 1994) rather than on multisensory integration, which was the focus of this study. Further research is needed to explore the relation between caregiver contingency and different aspects of the self.

It is important to note that our findings may not be entirely generalizable due to the use of touch in our fNIRS task. As noted by Ciaunica and Fotopoulou (2017), touch in experimental settings is inherently interpersonal and may have activated representations of social interactions in the infant, which could explain the observed influences of social interactions on neural activation. The relations may differ when considering self‐related tasks that do not involve touch but only visual‐proprioceptive contingencies. Only if these relations persist in such tasks can we confidently conclude that social interactions have an influence on the infant's self.

Another limitation of the present study is its correlational nature. Although we discussed the potential influences of mother‐infant interaction on infants’ multisensory integration, it is well possible that there is a bi‐directional relation between these variables. For example, infants with advanced multisensory integration abilities might appear to have a further developed self to the mother. This, in turn, might lead to the mother being less sensitive and providing less touch in the interaction, maybe to grant the infant more autonomy. Future research could use longitudinal designs to investigate the directionality of the relation.

## 6 Conclusion

Infants exhibit a neural prediction error when viewing a non‐contingent video of their own face being stroked, indicating an expectation for synchronous self‐related sensory information. However, infants show less of this prediction error when their mothers are more sensitive and touch them more during interactions. This suggests that social interactions influence the processing of multisensory contingencies, an important precursor of the infant's developing self.

## Ethics Statement

The study has been approved by the department's ethics committee and was conducted in accordance with the Declaration of Helsinki.

## Conflicts of Interest

The authors declare no conflict of interest.

## Supporting information



Supporting‐Information

## Data Availability

Raw data (without videos) and analysis scripts are available at https://osf.io/sng5e/.
