# Supplementary material for: Maternal Interaction Relates to Neural Processing of Self‐Related Multisensory Information in 5‐Month‐Olds
Source: Dev Sci. 2025 Mar 18;28(3):e70009. doi: 10.1111/desc.70009 (PMC11917191; doi:10.1111/desc.70009)
Supplement: Supplementary file 1 — Supporting‐Information [file DESC-28-e70009-s001.pdf]

Supplementary Material to “Maternal interaction relates to neural processing of self-related multisensory information in 5-month-olds”

Nina-Alisa Kollakowski, Carolina Pletti, Markus Paulus

Table S1

Post-Hoc Comparisons of HbO in Right ROI

Time Bin	Raw Time	*t*-Value	*p*-Value
**1**	**0-3 s**	**2.20**	**.03**
**2**	**3-6 s**	**2.34**	**.03**
**3**	**6-9 s**	**2.45**	**.02**
**4**	**9-12 s**	**2.13**	**.04**
**5**	**12-15 s**	**2.14**	**.04**
**6**	**15-18s**	**2.26**	**.01**

Note. Significant ($p < .05$) comparisons are presented in bold font.

Table S2

Post-Hoc Comparisons of HbO in Left ROI

Time Bin	Raw Time	*t*-Value	*p*-Value
1	0-3 s	0.03	.97
2	3-6 s	1.14	.26
3	6-9 s	1.61	.11
4	9-12 s	1.66	.10
5	12-15 s	2.04	.05
6	15-18s	1.93	.06

Note. Significant ($p < .05$) comparisons are presented in bold font.

Table S3

Linear Regression Results on the Differential Activation in the Left ROI

Variable	Estimate	SE	95% CI	t-Value	p-Value
Intercept	0.2885	0.6622	[-1.0520, 1.6290]	0.44	.67
Affective Touch	-0.0018	0.0077	[-0.0173, 0.0185]	-0.23	.82
All Touch	-0.0003	0.0023	[-0.0049, 0.0042]	-0.14	.89
Contingency	-0.1379	0.2503	[-0.6447, 0.3688]	-0.55	.59
Sensitivity	-0.0649	0.0548	[-0.1759, 0.0461]	-1.18	.24
Age	0.0001	0.0036	[-0.0072, 0.0075]	-0.04	.97

Table S4

Linear Regression Results on the Activation to the Non-Contingent Condition in the Right ROI

Variable	Estimate	SE	95% CI	t-Value	p-Value
**Intercept**	**1.8378**	**0.8406**	**[0.1313, 3.5443]**	**2.19**	**.04**
Affective Touch	0.0094	0.0094	[-0.0096, 0.0285]	1.01	.32
**All Touch**	**-0.0068**	**0.0027**	**[-0.0125, -0.0012]**	**-2.47**	**.02**
Contingency	0.0496	0.3031	[-0.5658, 0.6649]	0.16	.87
**Sensitivity**	**-0.1411**	**0.0663**	**[-0.2757, -0.066]**	**-2.13**	**.04**
Age	-0.0037	0.0045	[-0.0130, 0.0055]	-0.83	.42

Table S5

Linear Regression Results on the Activation to the Contingent Condition in the Right ROI

Variable	Estimate	SE	95% CI	t-Value	p-Value
Intercept	-0.2863	0.3974	[-1.0932, 0.5205]	-0.72	.48
Affective Touch	0.0046	0.0044	[-0.0044, 0.0136]	1.04	.31
All Touch	-0.0007	0.0013	[-0.0033, 0.0020]	-0.51	.61
Contingency	-0.0802	0.1433	[-0.3711, 0.2107]	-0.56	.58
Sensitivity	-0.0029	0.0313	[-0.0665, 0.0607]	-0.09	.93
Age	0.0014	0.0021	[-0.0030, 0.0057]	0.65	.52

Additional Analysis

We analyzed looking times in the fNIRS task. For this, we calculated the percent of time the infant looked at the screen for each trial, and compared mean looking times with a paired t-test. On average, infants looked at the screen for 73.15% (SD = 18.75) of time in the contingent condition and for 73.16% (SD = 18.86) of time in the non-contingent condition. These looking times were not significantly different from each other ($t(81) = 0.006$, $p = .99$). Correlations between average looking times and the respective average fNIRS activation in each condition are reported in Table S6.

Table S6

Correlations between fNIRS Signal and Looking Times

			Average Looking Time per Condition (%)		
	ROI	Condition	Non-Contingent	Contingent	Difference
fNIRS Activation (µMol)	Left	Non-Contingent	-0.21		
		Contingent		-0.08	
		Difference			0.39**
	Right	Non-Contingent	-0.37**		
		Contingent		0.09	
		Difference			-0.03

Note. *p < .05, **p < .01
